# Self-poisoning with paracetamol in England: short report of characteristics of individuals and their overdoses according to source of tablets

**DOI:** 10.1192/bjo.2024.740

**Published:** 2024-09-19

**Authors:** Fiona Brand, Elizabeth Bale, Apostolos Tsiachristas, Keith Hawton

**Affiliations:** Oxford Healthcare NHS Foundation Trust, Oxford, UK; Centre for Suicide Research, University of Oxford, Oxford, UK; Department of Psychiatry and Department of Primary Care Health Sciences, University of Oxford, Oxford, UK

**Keywords:** Self-harm, suicide intent, poisoning, paracetamol

## Abstract

Self-poisoning with paracetamol is the most frequently used overdose method in the UK. Psychosocial assessments were conducted by mental health clinicians with 127 consecutive individuals who presented with pure paracetamol overdoses to a large general hospital over 8 months, including asking about the source of the tablets and scoring the patients’ acts on the Beck Suicide Intent scale (BSI). Patients were predominantly female (86%) and young (79% aged 12–24 years). Most had used paracetamol which was available in the home (77%). Those who purchased paracetamol for the act took double the number of tablets compared with those who used paracetamol available in the home (37 *v.* 18), had higher suicidal intent (mean BSI: 11 *v.* 7) and more often required treatment with *N*-acetyl cysteine (71% *v.* 43%). These results highlight the need for safer home storage of paracetamol and consideration of reducing pack size limits on paracetamol that can be purchased.

## Background

Paracetamol overdose is the most commonly used method of intentional self-poisoning in the UK,^1^ with significant risk of death due to hepatotoxicity.^2^ Legislation introduced in September 1998 which restricted pack sizes for both pharmacy and non-pharmacy sales of paracetamol was associated with reduced deaths due to paracetamol overdose in both the short term and longer term.^3^ However, paracetamol overdose has remained extremely common. Based on data collected in three centres in England during 2004–2024, there were an estimated 50 000 hospital presentations by 36 000 individuals annually for overdoses of paracetamol alone,^1^ with many more involving paracetamol in compound form or consumed as part of multidrug overdoses or in combination with other self-harm methods. Paracetamol overdoses more often result in admission to a hospital bed than other types of overdose.^4^ They also involve higher hospital costs than self-poisoning with tranquilisers and mood stabilisers.^5^

Concerns have been raised that paracetamol has become increasingly easy to purchase, with some outlets having special offers, including in some cases for larger numbers of tablets than encouraged through the 1998 legislation. In previous interview studies of individuals who presented to hospital following self-poisoning with paracetamol, we found that in the majority of cases premeditation was very short, and the tablets taken were often those already present in the individual's households.^6,7^ Where tablets are purchased for the purposes of an overdose, it is likely that premeditation will be longer, and this might be associated with greater suicidal intent (wish to die) than is involved in probable impulsive overdoses of tablets already available in a household. Obtaining information about whether this is the case will be important for informing regulatory bodies about possible need for further initiatives to restrict availability of paracetamol to reduce the likelihood of self-poisoning. We have therefore conducted a study to investigate the sources of paracetamol taken in overdose, including whether this was the individual's household or whether the medication was purchased specifically for self-poisoning. We have also investigated whether the extent of patients’ suicidal intent, the sizes of overdoses and how often specific treatment for the overdose was provided varied according to the source.

## Method

The Oxford University Centre for Suicide Research has been collecting data on all persons presenting to the local general hospital with self-harm for many years.^8^ Using these data and in collaboration with members of the Emergency Department Psychiatric Service who assess patients presenting to the hospital following self-harm, we carried out an 8-month project (1 May to 31 December 2022) in which patients presenting to hospital with overdoses of paracetamol (not involving other drugs but potentially involving self-injury) were asked extra questions in addition to their usual psychosocial assessment regarding how they obtained the paracetamol. This included whether the tablets were purchased for the self-harm act or whether they were already available in the home. Most patients were also scored on the Beck Suicide Intent scale (BSI) (scores ranging from 0 to 38), with higher scores indicating that acts of self-harm were associated with a probable greater wish to die.^9,10^ This measure is usually completed as part of the routine psychosocial assessment. The clinicians also recorded whether patients had been treated with *N*-acetyl cysteine (NAC) while in hospital, a measure of the probable physical danger of the act. We only included in the study the first presentation for patients who presented with multiple episodes during the study period.

Descriptive statistical analysis was performed to calculate means and standard deviations of continuous variables and frequencies of categorical variables. Statistical differences between the purchase location groups were tested using the Kruskal–Wallis test and χ^2^-test, respectively. A regression analysis and its results are presented in the Supplementary file available at https://doi.org/10.1192/bjo.2024.740.

### Ethical approval

The Oxford Self-harm Monitoring System has approval from the National Health Service (NHS) Research Authority (NRES Committee South Central – Berkshire, 08/H0607/7) and also from the Health Research Authority Advisory Group under Section 251 of the NHS Act 2006 to process patient-identifiable information without explicit patient consent.

## Results

A total of 127 individuals presented to the hospital at least once during the 8-month study period having taken pure paracetamol overdoses. This included 19 acts where the overdose occurred in combination with some other form of self-harm, e.g. self-cutting. As shown in Table 1, the majority of the 127 individuals were female (86%) and young, with 59 (47%) aged 12–16 years and 41 (32%) aged 17–24 years. Nearly half were treated with NAC while in hospital (*n* = 62; 49%). Most individuals had self-harmed previously (*n* = 106; 84%). The mean BSI score was 7.7 (s.d. 6.1, *n* = 100), and the mean number of paracetamol tablets taken in overdose was 21.9 (s.d. 16.4, *n* = 123).

**Table 1:** Descriptive statistics by location of purchase

	Home only (*n* = 98)	Purchase only (*n* = 22)	Both (*n* = 7)	Total sample (*N* = 127)
Female at birth, *n* (%)	84 (86%)	19 (86%)	6 (86%)	109 (86%)
Age group, *n* (%)*				
12–16 years	51 (52%)	8 (36%)	0 (0%)	59 (47%)
17–24 years	26 (27%)	11 (50%)	4 (57%)	41 (32%)
25–93 years	21 (21%)	3 (14%)	3(43%)	27 (21%)
Received NAC, *n* (%)*	42 (43%)	15 (71%)	5 (71%)	62 (49%)
Previously self-harmed, *n* (%)	80 (82%)	20 (91%)	6 (86%)	106 (84%)
Combined with self-injury	14 (14%)	4 (19%)	1 (14%)	19 (15%)
BSI score, mean (s.d.) {median;IQR} [*n*] **	6.8 (5.0) {7;6} [75]	11.2 (9.1) {9;6} [19]	8.8 (3.7) {8.5;5} [6]	7.7 (6.1) {7;6} [100]
Tablets, mean number (s.d.) {median;IQR} [*n*] **	17.8 (12.0) {16;13} [95]	36.6 (22.7) {32.5;28} [22]	31.2 (16.0) {30;32} [6]	21.9 (16.4) {16;18} [123]

BSI, Beck Suicide Intent Scale (ranging from 0 to 38, with a higher score indicating a higher level of suicide ideation); NAC, *N*-acetyl cysteine.

* χ^2^-test *P* < 0.05; **Kruskal–Wallis test *P* < 0.05.

In more than three-quarters of cases, the paracetamol was obtained from the individuals’ homes (*n* = 98, 77%). In the remainder of cases, the individuals either purchased the paracetamol for the purpose of taking an overdose (*n* = 22, 17%) or, in a few cases, took an overdose of tablets obtained both through purchase and from the home (*n* = 7, 6%). There were no statistically significant differences between these three groups in terms of gender, previous incidence of self-harm and combination with self-injury. However, there were proportionally more individuals aged 12–16 years who used paracetamol available in the home (52%) compared with those who purchased it (35%) (none in this age group used paracetamol obtained from both their home and purchased for the act).

Individuals who purchased the paracetamol had significantly higher BSI scores than those who took paracetamol available in the home (mean 11.2, s.d. 9.1, *n* = 19 *v.* 6.8, s.d. 5.0, *n* = 75; Kruskal–Wallis test *P* < 0.05). Those who obtained the paracetamol from both sources had an intermediate mean BSI score between those of patients in the other two categories (mean 8.8, s.d. 3.7, *n* = 6). Considerably larger overdoses were taken by individuals who purchased the paracetamol (mean 36.6, s.d. 22.7) compared with those who took tablets available at home (mean 17.8, s.d. 12.0; Kruskal–Wallis test *P* < 0.05); those who obtained the paracetamol from both sources also took relatively large overdoses (mean 31.2, s.d. 16.0). The proportion of individuals who received NAC at the hospital was higher in the individuals who took paracetamol that they had purchased for the act (including those that also used paracetamol available in the home) (71%) compared with those who only took paracetamol available in their household) (43%) (χ^2^ *P* = 0.029).

## Discussion

In a typically young and predominantly female sample of consecutive individuals who presented to hospital having taken paracetamol overdoses, the vast majority (77%) used paracetamol which was already available in the home. This latter finding was somewhat greater than that in a previously interviewed sample from the same hospital, where 53% had taken paracetamol available in the home,^7^ but the participants in that study had all taken overdoses of more than 16 tablets. Our findings may have been influenced by the fact that the study was conducted during the latter part of the COVID-19 pandemic, at the outset of which there had been some increased purchasing of medication in anticipation of it being needed for treatment of COVID-19 symptoms.^11^ However, it was clear that overdoses of paracetamol purchased for the act involved greater suicidal intent and consumption of considerably larger numbers of tablets and more often resulted in treatment with NAC.

An implication of the findings of this study is that householders should wherever possible avoid having large amounts of paracetamol (or other analgesics) in the home, especially where a family member has a history of self-harm, and that such medication should be stored securely. In addition, further measures should be considered to further restrict sales of large amounts of paracetamol from pharmacies and other outlets. The current pack size limits for over-the-counter sales of paracetamol in the UK are the largest of European countries which have such legislation.^12^ Given that there continues to be a substantial number of deaths from paracetamol overdose in the UK, it would seem appropriate to consider reducing the current pack size limits for both pharmacy and non-pharmacy sales.

## Supporting information

Brand et al. supplementary material Brand et al. supplementary material

## Data Availability

Individual patient-level data are not available because of confidentiality concerns and data-sharing agreements currently in place.

## References

[1] Casey D, Geulayov G, Bale E, Brand F, Clements C, Kapur N, et al. Paracetamol self-poisoning: epidemiological study of trends and patient characteristics from the multicentre study of self-harm in England. J Affect Disord 2020; 276: 699–706. 32871703 10.1016/j.jad.2020.07.091

[2] Burns MJ, Friedman SL, Larson AM. Acetaminophen (Paracetamol) Poisoning in Adults: Pathophysiology, Presentation, and Evaluation. *Topic 340 Version 47.0.* UpToDate, 2024 (https://medilib.ir/uptodate/show/340).

[3] Hawton K, Bergen H, Simkin S, Dodd S, Pocock P, Bernwal W, et al. Long term effect of reduced pack sizes of paracetamol on poisoning deaths and liver transplant activity in England and Wales: interrupted time series analyses. BMJ 2013; 346: f403. 23393081 10.1136/bmj.f403 PMC3567205

[4] Tsiachristas A, Geulayov G, Casey D, Ness J, Waters K, Clements C, et al. Incidence and general hospital costs of self-harm across England: estimates based on the multicentre study of self-harm. Epidemiol Psychiatr Sci 2020; 29: e108. 32160934 10.1017/S2045796020000189 PMC7214546

[5] Tsiachristas A, McDaid D, Casey D, Brand F, Leal J, Park A, et al. General hospital costs in England of medical and psychiatric care for patients who self-harm: a retrospective analysis. Lancet Psychiatry 2017; **4**: 759–67. 10.1016/S2215-0366(17)30367-X PMC5614771 28890321

[6] Hawton K, Ware C, Mistry H, Hewitt K, Kingsbury S, Roberts D, et al. Paracetamol self-poisoning. Characteristics, prevention and harm reduction. Br J Psychiatry 1996; 168(1): 43–8. 8770427 10.1192/bjp.168.1.43

[7] Simkin S, Hawton K, Kapur N, Gunnell D. What can be done to reduce mortality from paracetamol overdoses? A patient interview study. QJM 2012; 105(1): 41–51. 21856743 10.1093/qjmed/hcr135

[8] Hawton K, Haw C, Casey D, Bale L, Brand F, Rutherford D. Self-harm in Oxford, England: epidemiological and clinical trends, 1996–2010. Soc Psychiatry Psychiatr Epidemiol 2015; 50(5): 695–704. 25488606 10.1007/s00127-014-0990-1

[9] Harriss L, Hawton K. Suicidal intent in deliberate self-harm and the risk of suicide: the predictive power of the Suicide Intent Scale. J Affect Disord 2005; 86(2–3): 225–33. 15935242 10.1016/j.jad.2005.02.009

[10] Beck AT, Schuyler D, Herman I. Development of suicidal intent scales. In The Prediction of Suicide (eds AT Beck, HL Resnik, Lettieri DJ): 45–56. Charles Press Publishers, 1974.

[11] Suda KJ, Kim KC, Hernandez I, Gellad WF, Rothenberger S, Cambell A, et al. The global impact of COVID-19 on drug purchases: a cross-sectional time series analysis. J Am Pharm Assoc (2003) 2022; 62(3): 766–74.e6. 35094929 10.1016/j.japh.2021.12.014 PMC8704785

[12] Morthorst BR, Erlangsen A, Nordentoft M, Hawton K, Hoegberg LCG, Dalhoff KP. Availability of paracetamol sold over the counter in Europe: a descriptive cross-sectional international survey of pack size restriction. Basic Clin Pharmacol Toxicol 2018; 122(6): 643–9. 29319222 10.1111/bcpt.12959

