# Supplementary material for: Self-poisoning with paracetamol in England: short report of characteristics of individuals and their overdoses according to source of tablets
Source: BJPsych Open. 2024 Sep 19;10(5):e155. doi: 10.1192/bjo.2024.740 (PMC11457225; doi:10.1192/bjo.2024.740)
Supplement: Brand et al. supplementary material [file S2056472424007403sup001.docx]

**Supplementary materials**

**Adjusted associations between location and number of tables and BSI score**

We performed a regression analysis to investigate the association of the source of paracetamol with the number of tablets and BSI score after adjusting for age, gender at birth, whether the self-poisoning was combined with self-injury, and whether the individual had a previous incidence of self-harm. For this, generalized linear mixed models (GLMs) with gamma distribution and log link were specified. The exponentiated coefficients in these models provided the ratio of means between the levels of dummy variables. As Figure 2 shows, individuals who purchased only had taken more than double (ratio of means 2.06, 95%CI: 1.54-2.76) the number of paracetamol tablets than those who had paracetamol at home.

Figure 1 Associations with number of paracetamol tablets

Figure 2 Associations with BSI score
